# The association between the built environment and intervention-facilitated physical activity: a narrative systematic review

**DOI:** 10.1186/s12966-022-01326-9

**Published:** 2022-07-14

**Authors:** Gavin R. McCormack, Michelle Patterson, Levi Frehlich, Diane L. Lorenzetti

**Affiliations:** 1 Department of Community Health Sciences, Cumming School of Medicine, University of Calgary, 3280 Hospital Drive, N.W., Calgary, Alberta T2N 4Z6 Canada; 2 Faculty of Kinesiology, University of Calgary, Calgary, Canada; 3 Faculty of Environmental Design, University of Calgary, Calgary, Canada; 4 Faculty of Sport Sciences, Waseda University, Shinjuku City, Japan; 5 Health Sciences Library, University of Calgary, Calgary, Canada

**Keywords:** Urban design, Interventions, Programs, Walkability, Physical activity, Exercise

## Abstract

**Background:**

A diverse range of interventions increase physical activity (PA) but few studies have explored the contextual factors that may be associated with intervention effectiveness. The built environment (BE) may enhance or reduce the effectiveness of PA interventions, especially interventions that encourage PA in neighbourhood settings. Several studies have investigated the effects of the neighbourhood BE on intervention-facilitated PA, however, a comprehensive review of evidence has yet to be conducted. In our systematic review, we synthesize evidence from quantitative studies that have examined the relationships between objectively-measured neighbourhood BE and intervention-facilitated PA in adults.

**Method:**

In October 2021, we searched 7 databases (Medline, CINAHL, Embase, Web of Science, SPORTDiscus, Environment Complete, and Cochrane Central Register of Controlled Trials) for English-language studies reporting on randomized and non-randomized experiments of physical activity interventions involving adults (≥18 years) and that estimated the association between objectively-measured BE and intervention-facilitated physical activity.

**Results:**

Twenty articles, published between 2009 and 2021, were eligible for inclusion in the review.

Among the 20 articles in this review, 13 included multi-arm experiments and 7 included single-arm experiments. Three studies examined PA interventions delivered at the population level and 17 examined interventions delivered at the individual level. PA intervention characteristics were heterogeneous and one-half of the interventions were implemented for at least 12-months (*n* = 10). Most studies were undertaken in North America (*n* = 11) and most studies (*n* = 14) included samples from populations identified as at risk of poor health (i.e., metabolic disorders, coronary heart disease, overweight, cancer, high blood pressure, and inactivity). Fourteen studies found evidence of a neighbourhood BE variable being negatively or positively associated with intervention-facilitated PA.

**Conclusion:**

Approximately 70% of all studies reviewed found evidence for an association between a BE variable and intervention-facilitated PA. The BE’s potential to enhance or constrain the effectiveness of PA interventions should be considered in their design and implementation.

**Supplementary Information:**

The online version contains supplementary material available at 10.1186/s12966-022-01326-9.

## Background

Regular physical activity (PA) protects against many chronic diseases [1, 2], functional limitations and disability [3, 4], and premature mortality [5]. However, despite these benefits, too many adults accumulate less than the required amount of PA needed to achieve optimal health [6]. Interventions are therefore needed to encourage adults to initiate and maintain regular PA. Two decades ago, Bauman et al. [7] acknowledged the importance of applying socioecological approaches to advancing the understanding of PA determinants, including the identification of factors that modify the effectiveness of PA interventions. The socioecological model provides a useful lens for conceptualizing how different factors interact at multiple levels (e.g., intra-individual, inter-individual, physical environment, policy, and culture) to influence PA [8–10] and is frequently used to inform studies investigating the built environment (BE) determinants of PA [10, 11].

The BE includes the human-modified physical surroundings and features (e.g., parks, streets, land uses destinations, connectivity, amenities, density, aesthetics, buildings, and transit) that people interact with to undertake their daily activities [12, 13]. Findings from reviews of cross-sectional and longitudinal studies [14–16] as well as qualitative studies [17] show that the neighbourhood BE can support and even inhibit PA. BE features including land use and destination mix and proximity, population and residential density, connectivity, and overall levels of walkability are consistently found to be associated with physical activity, and in particular with walking [14–17]. However, reviews investigating the links between the BE and PA to date have typically not focused on examining the influence of the BE on PA behaviour change resulting from participation in individual-targeted interventions (e.g., informational, or behavioural or social interventions). A PA supportive BE may be required to facilitate the success of individual-targeted PA interventions [18]. Likewise, individual-targeted PA interventions may be important for offsetting the negative impacts that unsupportive BEs might have on habitual physical activity. Qualitative findings from intervention studies suggest that the BE (e.g., access to nature, aesthetics, proximity to destinations, access to recreational facilities, pathways) is important for enabling changes in PA [19–21]. Quantitative findings from interventions studies however, appear to be mixed [22]. In a narrative review of eight quantitative studies, Zenk et al. [22] found weak evidence in support of a modifying effect of the BE on PA intervention effectiveness. The review revealed important insights, notably that few environmental indicators to date had been examined, many studies had methodological issues (e.g., low sample size limiting study power to detect effect modification), most studies included self-report measures of the BE, and that there was a need to explore the compatibility between interventions components and the available environmental opportunities and constraints.

Evidence regarding the effects of the neighbourhood BE on intervention-facilitated PA (i.e., physical activity outcomes resulting from participation in an intervention) have not been comprehensively examined and summarized. Moreover, advancing knowledge with regard to the impact of the neighbourhood BE on intervention-facilitated PA may contribute to improving intervention delivery and effectiveness [23] and addressing the calls made almost two decades ago for rigorous evidence on person-environment interactions in relation to PA (i.e., integration of individual and environmental level factors) [7, 24]. Therefore, the aim of our study was to undertake a systematic review of quantitative studies that have examined the relationships between objectively-measured neighbourhood BE and intervention-facilitated PA in adults.

## Method

### Search strategy and study selection

Details of the protocol for this systematic review were registered on PROSPERO (CRD42021297191) [25]. We undertook a systematic literature review in accordance with the Preferred Reporting Items for Systematic Reviews and Meta-analysis (PRISMA) guidelines [26] (Supplement 1: PRISMA Checklist). We searched seven databases (MEDLINE, CINAHL, Embase, Web of Science, SPORTDiscus, Environment Complete, and the Cochrane Central Register of Controlled Trials) for English-language studies that included both PA interventions and measures of the BE. We imposed no constraints on year of publication but did restrict the search to studies involving adult populations (≥18 years of age). Within each database, a comprehensive list of keywords and subject terms associated with the BE, PA, intervention, and adult populations was searched within titles and abstracts (Table 1). In collaboration with a University of Calgary health sciences librarian, we develop the search strategy, including the list of search terms. The search was finalized October 5, 2021. One author (MP) screened all abstracts and a second author (LF) with research experience in the topic area, blindly screened a random sample of abstracts as a quality check. The two authors reached agreement regarding which articles were eligible to undergo full-text review. Three authors (MP, LF, and GRM) read all full-text articles and reached consensus regarding the final list of articles that would be included the review.

**Table 1:** List of search terms and subject headings used in the systematic review

	Neighbourhood built environment	Physical Activity	Intervention	Adult
Terms	built environment*; physical environment*; objective environment*; environment*; design; urban form*; urban design*; neighborhood*; neighbourhood*; GIS; geographic information system*; open space*; greenspace*; green space*; pedestrian-friendly; walkability; walkable; bikeability; bikeable; cyclability; cyclable; park*; park; urban planning; street	physical activit*; exercis*; recreation*; leisure; sport*; bik*; bicycl*; cycling; jog*; run*; stroll*; walk*; active transport*; pedestrian*	Intervention*; campaign*; program*	Adult*; senior*; elderly
Subject headings**	Built environment; Environment design; Environmental planning; Neighborhood	Exercise; Recreation; Physical activity	Health promotion; Health program; Intervention study; Intervention studies	Aged; Adult; Male; Female; Older people

*Different variations of the terms used in search

**Subject headings were specific to the database searched and not all listed subject headings were available for all databases

### Eligibility criteria

Eligible articles met six inclusion criteria, including: 1) a quantitative or mixed methods study; 2) an intervention implemented with the primary aim of increasing PA; 3) a repeated measures study design with an intervention treatment group with or without a comparison group (i.e., quasi-experiments, natural experiments, randomized controlled trials, cohort studies, or repeated cross-sectional designs); 4) an objective measure of the BE (e.g., Geographical Information Systems (GIS), desktop mapping, and street audits); 5) a self-report (e.g., questionnaire) or objective (e.g., accelerometer) measure of PA as an intervention outcome, and; 6) an estimate of association between the BE and PA accumulated while participating in the intervention (i.e., intervention-facilitated PA). Intervention studies that combined treatment and comparison groups when estimating the association between the BE and PA (i.e., pooled estimate) were included in the review only if the comparison group received a sub-set of intervention components that were also offered to the treatment group (e.g., partial intervention). We excluded studies involving interventions that modified the BE. We also excluded commentaries, editorials, books, literature reviews, conference proceedings or abstracts, qualitative research articles, and grey literature.

### Data extraction and reporting

A data extraction form was created to record study specific details regarding the study and sampling design, sample characteristics, intervention characteristics, objective BE measures, PA measures, and findings in relation to the BE effect on intervention-facilitated PA. We also extracted information about intervention implementation including type and length of treatment, whether participants received instructions to undertake their own versus attend formal exercise sessions, received counselling, including but not limited to, motivational interviews and follow-up calls, written or verbally-delivered educational or informational materials related to PA behaviour or benefits, and were offered opportunities for group-based PA. MP completed the data extraction, and the co-authors (LF and GRM) verified the recorded information for accuracy. Discrepancies were addressed via consensus. Extracted data were summarized and tabulated. Several studies included findings related to self-report BE measures however, these were not included in the review. Several studies included results that combined treatment and comparison groups to obtain an overall estimate of association between the BE and PA however, these results were only reported if the comparison group received a subset of intervention components that coincided with those received by the treatment group (i.e., full versus partial intervention).

The included studies were heterogeneous in terms of their design and methods and not adequate for meta-analysis, thus we undertook a narrative (or descriptive) synthesis of findings [27]. Key findings from studies that were estimated from multivariate analysis (i.e., covariate-adjusted) were summarized when available. Studies were included if they: 1) tested for an interaction between a BE variable and intervention arm, and or; 2) estimated the association between a BE variable and intervention-facilitated PA within a study arm (i.e., within group effect) or within a single group receiving the intervention (e.g., one-group experimental design or where intervention arms were combined).

**Fig. 1:**
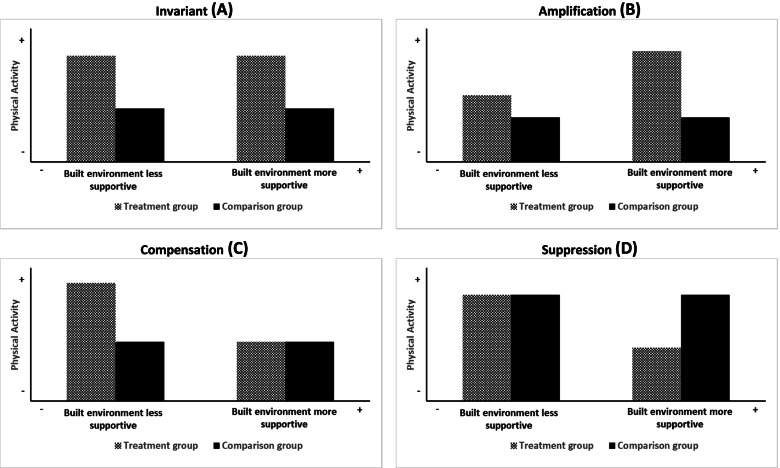
Conceptual framework and graphical display of potential mechanisms underlying the interrelationships between the built environment, intervention, and intervention-facilitated physical activity. Invariant **A** The built environment has minimal influence on the effects of the physical activity invention. The intervention increases physical activity to a similar degree in less and more supportive built environments. Amplification **B** A more supportive built environment amplifies the effects of the physical activity intervention. The intervention increases physical activity to a greater degree in a more supportive built environment than in a less supportive built environments. Compensation **C** A more supportive built environment has minimal influence on the effects of the physical activity intervention but the intervention is effective in increasing physical activity in a less supportive built environment. Suppression **D** A less supportive built environment has minimal influence on the effects of the physical activity intervention but the effects of the intervention on physical activity are constrained in a more supportive built environment. Note that built environment-intervention interactions could also include the combination of compensation-suppression mechanism (i.e., the intervention is effective in increasing physical activity in a less supportive built environment while the effects of the same intervention on physical activity are constrained in a more supportive built environment)

To assist in the interpretation of the findings of each study, we generated hypothetical scenarios to assist in explaining the BE’s influence on intervention-facilitated PA (Fig. 1). We identified four potential pathways by which the BE might modify the effects of the intervention on PA: 1) invariant; 2) amplification; 3) compensation, and; 4) suppression. These pathways were informed by and adapted from literature originally aimed at understanding potential mechanisms underlying gene-environment [28, 29] and ecological [30] interactions.

*Invariant* associations included those where the BE had minimal impact on an intervention that was effective for increasing PA. For example, an intervention involving the delivery of exercise programs in local parks may be equally effective for increasing physical activity regardless of a neighbourhood’s walkability. *Amplification* reflected associations where a supportive BE appeared to augment the effectiveness of the PA intervention. For example, a neighbourhood with infrastructure that makes walking convenient and enjoyable such as well-connected sidewalk and pathway networks, pedestrian amenities, and integrated nature spaces and parks may further enhance the effectiveness of an intervention targeting leisure walking. *Compensation* reflected associations where the intervention is more effective for increasing PA among those residing in less supportive BEs. For example, participants exposed to an intervention designed to encourage active transport (e.g., personalized trip planning) may be more responsive to the intervention if they reside in neighbourhoods that are less supportive of this behaviour. Alternatively, those exposed to the same intervention but residing in neighbourhoods that already support active transport may be less responsive to the intervention. *Suppression* reflected associations whereby a more supportive BE negatively impacted an intervention that is generally considered effective for increasing PA (i.e., constraining or reducing PA among adults residing in more supportive BEs). Suppression could also reflect when an effective intervention implemented in a less supportive BE results in a reduction in PA. Suppression might suggest poor fit or mismatch between intervention components and the BE. For single arm studies (no control), BE suppression and compensation are more difficult to determine as its possible that a single negative association between a BE characteristic and intervention-facilitated PA could reflect compensation when the BE characteristics is less favorable and suppression when the BE characteristics is more favorable.

### Assessment of study quality

Study quality was assessed independently by MP and LF using a previously validated check-list tool for assessing methodological quality of randomized and non-randomized quantitative studies [31]. The checklist included 27 questions (yes: score = 1 or no: score = 0) divided into five components including reporting quality (maximum score = 10), external validity (maximum score = 3), internal validity-bias (maximum score = 7), internal validity-confounding and selection bias (maximum score = 6), and statistical power (maximum score = 1). Component and overall study quality scores were estimated. We did not use quality scores to prioritize study findings nor did we exclude low quality studies.

## Results

### Study selection

**Fig. 2:**
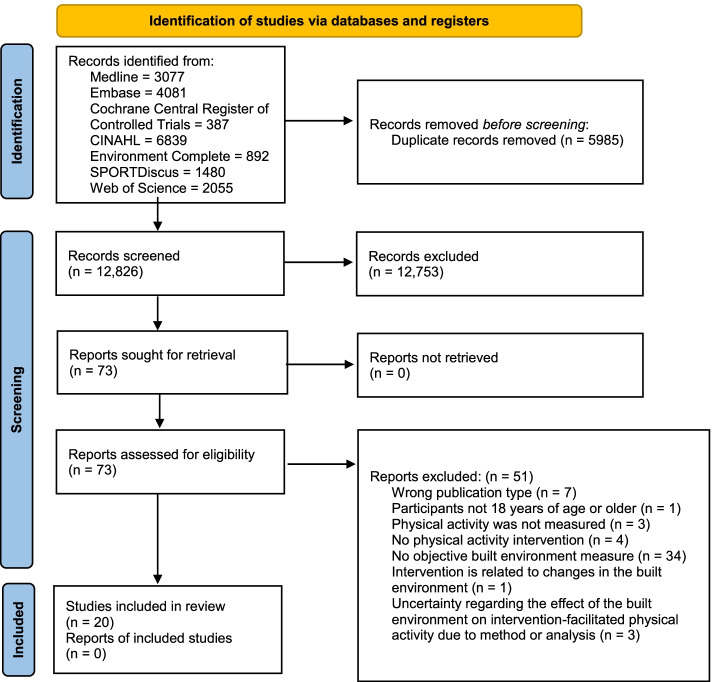
PRISMA 2020 flow diagram of article search and selection for the review

The database search resulted in 12,826 non-duplicate records (Fig. 2). One author (MP) screened all abstracts and identified 83 eligible records. A second author (LF) with research experience in the topic area, blindly screened a random sample of 390 records in addition to the 83 records (total *n* = 473; approximately 4% of all non-duplicate records) identified by MP to estimate inter-rater agreement (percentage of overall agreement = 91%). Reaching consensus, MP and LF identified 73 records to undergo full-text review. The full-texts for all 73 records were obtained and reviewed in duplicate by MP and LF who applied the inclusion and exclusion criteria. Consensus between MP and LF and a co-author (GRM) resulted in 20 full-text articles identified as eligible for inclusion in the review.

### Study sample characteristics

Of the 20 articles included in this review, a higher number included studies undertaken in the USA (*n* = 8) followed by the UK (*n* = 4) and Canada (*n* = 3) with two studies being conducted in Japan, and one study being conducted each in Australia, Spain, and Chile. Studies were published between 2009 and 2021 (Table 2). Most studies (*n* = 16) recruited samples from populations with a known health risk, including metabolic disorders (e.g., diabetes risk, metabolic syndrome) [32, 35], coronary heart disease risk factors [42], overweight or obesity [36], history of cancer [40], high blood pressure [51], and sedentary behaviour or physical inactivity [34, 43, 44, 46, 48] or a combination of risk factors [33, 37, 39]. Six studies included samples from general (non-clinical) populations [38, 41, 45, 47, 49, 50].

**Table 2:** Summary of findings for studies testing the effect of the objective built environment on intervention-related physical activity outcomes (*n* = 20)

Author (year)	Study location	Sample design	Study design	Intervention details	Built environment measures included in analysis	Physical activity measures examined in relation to the built environment	Summary of key findings [***Potential mechanism***]^**a**^
**Carter et al. (2017)** **[** 32**]**	Leicester City/ Leicestershire County, UK (urban & rural)	At-risk of type 2 diabetes mellitus, 18–74 years Non-probability sample Analytical *n* = 706	Two-arm RM RCT Arms: 1. Intervention 2. Standard care	Type: Individual *(Walking away from diabetes)* Exercise sessions: No Counselling: Yes Awareness/education: Yes Group-based activities: Yes Length: 3 sessions (at baseline, 12 & 24 months)	800 m network buffer around home Walkability index (walkable area, road density, footpath density, junctions, cul-de-sacs, & connected node ratio).	Accelerometer Measured at baseline, 12, 24, & 36 months Domain(s): Non-specific PA type(s): MVPA Steps	“Arm x walkability index” interaction significantly associated with change in total steps & purposeful steps at 36-months. However, stratified analysis found no significant associations between *walkability* with change in total steps in the intervention (b = − 63.38, *p* > .05) or control (b = 19.68, *p* > .05) arms nor purposeful steps in the intervention (b = 42.97, *p* > .05) or control (b = 25.77, *p* > .05) arms. [***Invariant*** **]**
**Colom et al. (2020)** **[** 33**]**	Palma de Mallorca, Spain	Overweight/obese with metabolic syndrome, 55–75 years Non-probability sample Analytical *n* = 228	Two-arm RM RCT Arms: 1.Full intervention 2.Partial intervention	Type: Educational with individual & group sessions (*PREDIMED-Plus*) Exercise sessions: No Counselling: No Awareness/education: Yes Group-based activities: Yes Length: 12 months (12 × 1 hr. one-on-one sessions, 12 telephone calls, 3 × 1 hr. group sessions)	500 m/1000 m network sausage buffer around home Walkability index; residential density; intersection density; & land use mix	Accelerometer & self-reported Measured at baseline, 6 & 12 months Domain(s): Leisure/recreation Non-specific PA type(s): MVPA Walking	“Arm x walkability” interaction significantly associated with change in accelerometer-PA. *Walkability* associated with daily minutes of accelerometer-PA in intervention arm (b = 6.36, *p* < .05) but not control arm (b = 0.10, *p* > .05).*[* ***Amplification*** *]* *Residential density* associated with daily minutes of accelerometer-PA in intervention arm (b = 5.07, *p* < .05) but not control arm (b = 1.95, *p* > .05).*[* ***Amplification*** *]* *Intersection density* associated with daily minutes of accelerometer-PA in intervention arm (b = 6.86, p < .05) but not control arm (b = 1.38, *p* > .05).*[* ***Amplification*** *]* *Land use mix* not associated with accelerometer-PA in either intervention or control arm. *[* ***Invariant*** *]*
**Goyder et al. (2014)** **[** 34**]**	Sheffield, UK	Residents of deprived neighbourhoods (40–64 years), not achieving recommended levels of activity & wishing to have more support for PA Non-probability sample Analytical n: mini booster *n* = 47, full booster *n* = 52, control = 61	Three-arm, parallel arm RM RCT Arms: 1. Full intervention 2. Partial intervention 3. Control	Type: PA consultations, using motivational interviewing (*Sheffield Physical Activity Booster Trial*) Exercise sessions: No Counselling: Yes Awareness/education: Yes Group-based activities: No Length: Brief (motivational DVD mailed to participants) & 2 follow up monthly phone calls	Postcode centroid Pedestrian access to municipal parks, swimming pools, & gyms using network distance analysis	Accelerometer Measured at baseline, 3 & 9 months Domain(s): Non-specific PA type(s): Total PA	No significant interactions between any BE variables & intervention arm in relation to energy expenditure at 3-months (9-month results not tested) *[* ***Invariant*** *]*
**Hays et al. (2016)** **[** 35**]**	Indianapolis, Indiana, USA	Inner city with risk of diabetes, ≥18 years Non-probability sample Analytical *n* = 216	Two-arm RM RCT Arm: 1. Full intervention 2. Partial intervention	Type: Brief counselling plus a grouped-based diabetes prevention program (*RAPID*) Exercise sessions: No Counselling: Yes Awareness/education: Yes Group-based activities: Yes Length: 24 months (16 × 60–90 min sessions)	Walkability index (Walk Score®) & Normalized Differential Vegetation index (NDVI) at census tract-level	Accelerometer Measured at baseline, 6, 12, & 24-months Domain(s): Non-specific PA type(s): MVPA	No “BE x intervention interaction” tested. Separate estimates for intervention arm not presented (i.e., full & partial intervention pooled estimates). Higher *NDVI* was associated with increases in MVPA over time (b = − 115.03, *p* < .05). *[* ***Amplification*** *]*
**Kerr et al. (2010)** **[** 36**]**	San Diego County, USA	Overweight women (18–55 years) & men (25–55 years) Non-probability sample Analytical n: men = 89/ women = 83)	Two-arm pre-post RCT Arms: 1. Intervention 2. Standard care (some differences in interventions delivered to men vs. women)	Type: Computer-facilitated personalized action plan with brief counselling (*PACE*) – intervention delivery differed by sex. Exercise sessions: No Counselling: No Awareness/education: Yes Group-based activities: No Length: 12 months	1600 m network buffer around home. Walkability index (residential density, intersection density, land use mix, retail floor area ratio).	Self-reported Measured at baseline & 12 months. Domain(s): Non-specific PA type(s): Walking	Men: “Time x arm x walkability” interaction significantly associated with change in daily walking duration. Intervention arm in *low walkable neighbourhoods* increased walking by 29-min/day *[* ***Compensation*** *]* versus intervention arm in *high walkable neighbourhoods* where walking decreased by 10-min/day [***Suppression***]. No change in walking for control arm regardless of neighbourhood walkability Women: No significant “time x arm x walkability” interaction. *[* ***Invariant*** *]*
**King et al. (2017)** **[** 37**]**	4 sites: Texas, California, Pennsylvania, & North Carolina, USA	Sedentary, at major risk for mobility disability, 70–89 years. Non-probability sample Analytical *n* = 400	Two-arm pre-post RCT Arms: 1. Intervention 2. Health education	Type: Centre-based (supervised) & home-based aerobic activities, strength training, flexibility, & balance exercises (*LIFE-Pilot*) Exercise sessions: Yes Counselling: Yes Awareness/education: Yes Group-based activities: Yes Length: 12 months (adoption phase: 2 months × 3 × 40-60 min session/wk.; transitional phase: 4 months 2 × 40-60 min session/wk.; maintenance phase: 6 months x optional 1–2 × 40-60 min session/wk)	250 m buffer around home. Walkability index (residential density, intersection density, land use mix, retail to floor area ratio)	Self-reported Measured at baseline & 12-months Domain(s): Leisure/recreation Transport/errands PA type(s): Walking	No significant “arm x walkability” interaction associated with exercise/leisure walking. *[* ***Invariant*** *]* “Arm x walkability” interaction approached significance for weekly duration of walking for errands (*p* = .07). In low walkable neighbourhoods, walking for errands decreased by 15.4 min/week in intervention arm and increased by 8.7 min/week in health education arm (*p* < .01 for between arm difference). No between arm differences in walking for errands among high walkable neighbourhoods. However, results figures showed similar declines in walking for errands in intervention arm for both low and high walkable neighbourhoods. ***[Invariant]***
**Lee et al. (2012)** **[** 38**]**	Houston & Austin, USA	African American, Hispanic or Latino women, 25–60 years Non-probability sample Analytical *n* = 309	Two-arm pre-post RCT Arms: 1 PA Intervention 2. Nutrition intervention	Type: Group/team facilitated session & activity with personalized behaviour change plans (*Health is Power*) Exercise sessions: Yes Counselling: Yes Awareness/education: Yes Group-based activities: Yes Length: 6 months (6 group sessions)	400 m radial buffer around homes. Pedestrian environmental data scan (audit of street segments): features & facilities related to walking & cycling (e.g., indicators of land use, connectivity, lighting, articulation, road attributes, safety attractiveness, sidewalks, & amenities)	Self-reported & accelerometer Measured at baseline & 6-months Domain(s): Non-specific PA type(s): MVPA (accelerometer) Total PA (self-report) Walking (self-report)	“Arm x BE” interaction significantly associated with post-intervention self-reported walking (number of *traffic control devices* & number of *amenities*) & total PA (number of *crossing aids* & number of *amenities*), but not with accelerometer-PA. Positive association between number of *traffic control devices* and post walking in PA group relative to a negative association in nutrition group (b = − 0.80, *p* < .05) [***Amplification in PA arm***] Positive association between number of *crossing aids* and post total PA in PA group relative to a negative association in nutrition group (b = − 0.46, *p* < .05) [***Amplification in PA arm***] Negative association between number of *amenities* and post walking in PA group relative to a positive association in nutrition group (b = 0.45, *p* < .05) [***Suppression in PA arm***] Negative association between number of *amenities* and post total PA in PA group relative to a positive association in nutrition group (b = 0.33, *p* < .05) [***Suppression in PA arm***]
**Lo et al. (2019)** **[** 39**]**	Montana & New York, USA (rural)	Sedentary overweight women, ≥40 years. Non-probability sample Analytical *n* = 151	Two-arm pre-post clustered RCT Arms: 1. Full intervention 2. Partial intervention	Type: Multi-component, class-based exercise with motivational interviewing & awareness raising (*Strong Hearts, Healthy Communities*) Exercise sessions: Yes Counselling: Yes Awareness/education: Yes Group-based activities: Yes Length: 6 months (2 sessions/wk)	Walkability index (Walk Score®)	Accelerometer Measured at baseline & 6 months Domain(s): Non-specific PA type(s): MVPA	“Arm x Walk Score®” interaction not significantly associated with post-intervention daily minutes of MVPA or percent of MVPA. However, in neighbourhoods with a *Walk Score®* = 0, change in minutes (11.7 min/day) & percent (1.5%) of MVPA was significantly (*p* < .05) higher in intervention arm than change in minutes (−0.8 min/day) & percent (− 0.1%) of MVPA in control arm. No other between arm differences found for other Walk Score® levels. ***[Likely Compensation]***
**McGowan et al. (2017)** **[** 40**]**	Edmonton, Canada	Prostate cancer survivors, ≥18 years Probability sample Analytical *n* = 165	Three-arm RM RCT Arms: 1. Intervention (self-administered implementation intention; SAII), 2. Intervention (telephone-assisted implementation intention; TAII) 3. Brief information (PA factsheet: SPAR)	Type: Individual-based, no group sessions, information provided, no PA or exercise sessions (*PROMOTE*). Exercise sessions: No Counselling: Yes Awareness/education: Yes Group-based activities: No Length: Single session	500 m/1000 m network buffers around homes. Walkability index (intersection density, residential density, & land use mix). Count of sport complexes (included facilities for indoor/outdoor activities)	Self-reported Measured at baseline, 1 & 3 months Domain(s): Leisure/recreation PA type(s): Total PA	“Time x arm x BE” interactions not significantly associated with minutes of self-reported weekly PA nor achievement of 150 minutes/week. *[* ***Invariant*** *]*
**Michael & Carlson (2009)** **[** 41**]**	Portland, USA	Mobility non-restrictive, cognitively sound adults not enrolled in formal exercise programs, ≥65 years. Probability sample Analytical *n* = 582	Two-arm RM clustered RCT Arms: 1. Intervention 2. Education only	Type: Led walking groups (*SHAPE*) Exercise sessions: Yes Counselling: No Awareness/education: No Group-based activities: Yes Length: 6 months (3 sessions/wk)	400 m/800 m radial buffer around home. Walkability index (e.g., sidewalk coverage, connectivity, public transportation access, distribution of parks/green space, & level of automobile traffic volume).	Self-reported Measured at baseline, 3, & 6-months Domain(s): Non-specific PA type(s): Walking	“Arm x walkability” interaction not significantly associated with weekly minutes of brisk walking measured at 6-months. *[* ***Invariant*** *]*
**Riley et al. (2013)** **[** 42**]**	Ottawa, Canada	≥1 modifiable CHD behavioral risk factor, 20–80 years, with relative hospitalized for CHD in past 5-years Non-probability sample Analytical *n* = 230	Two-arm pre-post RCT. Arms: 1. Intervention 2. Standard care	Type: Individual counselling sessions with a personalized behavior change plan (*Family Heart Health*) Exercise sessions: No Counselling: Yes Awareness/education: Yes Group-based activities: No Length: 12 weeks (1 in-person counselling session, followed by 12 weekly telephone calls)	Spatially defined neighbourhoods Walkability index (Walk Score®) & Walkability index (intersection density, residential density, retail floor area ratio & land use mix)	Self-reported Measured at baseline & 12-weeks Domain(s): Leisure/recreation PA type(s): MVPA	“Arm x Walk Score®” interaction not significantly associated with meeting the PA guidelines at 12 weeks (≥150 min/week of MVPA). *[* ***Invariant*** *]* “Arm x walkability index” interaction not significantly associated with meeting the PA guidelines at 12-weeks (≥150 min/week of MVPA). *[* ***Invariant*** *]*
**Robertson et al. (2012)** **[** 43**]**	Glasgow, Scotland, UK	Insufficiently active, 18–65 years Non-probability sample Analytical *n* = 45	Two-arm RM RCT Arms: 1. Full intervention 2. Partial intervention	Type: Pedometer facilitated individualized walking program (*Walking for Well-Being in the West*) Exercise sessions: Yes Counselling: Yes Awareness/education: Yes Group-based activities: No Length: 3 months	400 m radial buffer around home but also tested 1600 m. Street audits using the Scottish Walkability Assessment Tool (SWAT) & GIS measures (green space & recreation facilities, commercial & residential land use mix, dangerous & busy roads, pathway features other than safety, pathway safety features, roads & bus stops indoor fitness facilities & traffic calming features, traffic signals & pedestrian signage). Factors created.	Pedometer Measured at baseline, 3, 6, & 12-months Domain(s): Non-specific PA type(s): Steps	Separate estimates for intervention arm not presented (i.e., full & partial intervention pooled estimates) *Dangerous & busy roads factor* was negatively associated with step count at 3-months (b = −0.28, *p* < .05) and 6-months (b = − 0.31, *p* < .05). [***Amplification***] *Commercial & residential land use factor* was positively associated with step counts at 6-months (b = 0.40, *p* < .05) and 12-months (b = 0.30, *p* = .05) *[* ***Amplification*** *]* *Traffic signals & pedestrian signage factor* was negatively associated with step count at 6-months (b = − 0.30, *p* < .05). [***Amplification***] *Indoor fitness facilities & traffic calming features factor* was positively associated with step counts at 6-months (b = 0.27, *p* < .05). *[* ***Amplification*** *]* *Green space & recreation facilities factor* was negatively associated with step counts at 12-months (b = − 0.34, *p* < .05) *[* ***Suppression*** *]* *Pathway features other than safety factor*, *Pathways safety features factor*, & *Roads & bus stop factor* not associated with steps at any period. *[* ***Invariant*** *]*
**Zenk et al. (2009)** **[** 44**]**	Chicago, USA	Sedentary African American women, no signs/symptoms of CVD, 40–65 years Non-probability sample Analytical *n* = 252	Two-arm post measure quasi-experiment Arms: 1. Full intervention 2. Partial intervention	Type: Walking program, including walking prescription, plus motivational workshops (*The Women’s Walking Program*) Exercise sessions: Yes Counselling: Yes Awareness/education: Yes Group-based activities: Yes Length: 12 months (24 week adoption phase including weekly motivation workshops & 24 week maintenance phase including weekly or bi-weekly telephone calls)	1600 m radial buffer around home. Walkability index (land use mix, street connectivity, housing unit density, public transit stop density); Aesthetics; physical deterioration; industrial land use; availability of outdoor facilities & spaces; percentage of recreational open space area; indoor facilities; presence of public recreation center with indoor track or treadmill & shopping mall.	Self-reported & heart rate monitoring Measured post-intervention (i.e., accumulated PA) Domain(s): Non-specific PA type(s): Prescribed walks completed	No significant interactions between arm & any BE variables related to adherence to the program during the adoption phase. *[* ***Invariant*** *]* Separate estimates for intervention arm not presented (i.e., full & partial intervention pooled estimates). Presence of either a public recreation center with treadmill or indoor track or indoor shopping mall within 5 miles was associated with higher walking adherence relative to no facility present (b = 0.39, *p* < .05). ***[Amplification]*** Walkability not significantly associated with walking adherence (b = − 0.12, *p* > .05). ***[Invariant]***
**Barnes et al. (2013)** **[** 45**]**	**Perth, Australia**	**General population, 20–54 years** **Probability sample** **Analytical n: pre-interventio** ***n*** **= 466, post-intervention = 360)**	**One-arm pre-post quasi-experiment (repeated cross-sectional surveys)** **All participants exposed to intervention**	**Type: Mass media (** ***Find Thirty Every Day*** **)** **Exercise sessions: No** **Counselling: No** **Awareness/education: Yes** **Group-based activities: No** **Length: 12 months**	**1600 m network buffer around home** **Recreational walkability index (dwelling density, street connectivity, & land use mix)**	**Self-reported** **Measured at pre & post intervention** **Domain(s):** **Transport/errands** **Non-specific** **PA type(s):** **Total PA** **Walking**	“Time x walkability” interaction not significantly associated with any PA outcomes. However, adults from *low walkable neighbourhoods* were less likely to report any transport walking post vs. pre intervention (OR = 0.7, *p* < .05) while there was no significant change among those in high walkable neighbourhoods (OR = 0.9, *p* > .05). ***[Likely suppression]*** Adults from *low walkable neighbourhoods* were more likely to report sufficient PA post vs. pre intervention (OR = 1.4, *p* < .05) while there was no significant change among those in high walkable neighbourhoods (OR = 1.6, *p* > .05). ***[Likely invariant]***
**Consoli et al. (2020)** **[** 46**]**	Calgary, Canada	Inactive, ≥18 years Non-probability sample Analytical n = 466	One-arm pre-post quasi-experiment All participants received intervention	Type: Internet-facilitated pedometer intervention (*UWALK*) Exercise sessions: No Counselling: No Awareness/education: Yes Group-based activities: No Length: 3 months	Walkability index (Walk Score®)	Pedometer Measured post-intervention (i.e., accumulated PA) Domain(s): Non-specific PA type(s): Steps	Walk Score® was not associated with count of days steps recorded (IRR = 1.0, *p* > .05), achievement of 10,000 steps/day (IRR 1.0, *p* > .05), or daily steps during the intervention (b = 4.0, *p* > .05). *[* ***Invariant*** *]*
**Garmendia et al. (2013)** **[** 47**]**	Santiago, Chile	Residents of low socioeconomic county that had more than 400 persons (65–67.9 yrs) Non-Probability sample Analytical *n* = 996	Original design included a multi-arm RM experiment but results for only one arm included in the analysis. All participants received a PA intervention	Type: Physical activity group exercise sessions Exercise sessions: Yes Counselling: No Awareness/education: No Group-based activities: Yes Length: 24 months (2 × 1 hr. sessions/wk)	Predefined neighbourhoods in Observatory of the Government of Chile Distance from home to the PA center (where activities were undertaken), and well-kept community green areas (m^2^/inhabitant)	Recorded Measured at 24 months Domain(s): Leisure/recreation PA type(s): Attendance at PA sessions (≥24 sessions)	*Distance from home to the PA center* not significantly associated with adherence to intervention (OR = 1.0, *p* > 0.2). *[* ***Invariant*** *]* Adherence was positively associated with *neighbourhood area of well-kept green spaces* (OR = 1.2, *p* < .01) *[* ***Amplification*** *]*
**Goyder et al. (2016)** **[** 48**]**	Sheffield, UK	Sedentary, from SES deprived neighbourhoods, 40–64 years Non-probability sample Analytical *n* = 941	One-arm pre-post quasi-experiment All participants received intervention	Type: PA consultations, using motivational interviewing (Sheffield Physical Activity Booster Trial) Exercise sessions: No Counselling: Yes Awareness/education: Yes Group-based activities: No Intervention length: Brief (motivational DVD mailed to participants) & 2 follow up monthly phone calls	Shortest pedestrian network distance to: greenspace, gyms & pools from home.	Self-reported Measured at baseline & 3-months Domain(s): Non-specific PA type(s): Total PA	BE variables were not significantly (*p* > .05) associated with the likelihood of increasing PA due to intervention (gym: OR = 1.0; greenspace: 0.9; pool: OR = 0.9). *[* ***Invariant*** *]*
**Hino et al. (2019)** **[** 49**]**	Yokohoma City, Japan	General population, ≥40 years Probability sample Analytical *n* = 2023	One-arm pre-post quasi-experiment All participants received intervention	Type: Pedometer facilitated walking program (*Yokohama Walking Point Program*) Exercise sessions: No Counselling: No Awareness/education: Yes Group-based activities: Yes Length: ~ 30 months	Distance to nearest railway station from center of each participant’s neighbourhood, & neighbourhood bus stop density.	Self-reported Measured post-intervention (i.e., accumulated PA) Domain(s): Non-specific PA type(s): Relative change in daily step count due to program	*Bus stop density* was positively associated with an increase in step counts due to the program (b = 0.04, *p* < .01). *[* ***Amplification*** *]* *Distance to nearest railway station* was not associated with an increase in step counts due to the program (b = 0.02, p < .05). ***[Invariant]***
**Hino et al. (2021)** **[** 50**]**	Yokohoma City, Japan	General population, ≥40 years Probability sample Analytical *n* = 47,233	One-group pre-post quasi-experiment All participants received intervention	Type: Pedometer facilitated walking program (*Yokohama Walking Point Program*) Exercise sessions: No Counselling: No Awareness/education: Yes Group-based activities: Yes Length: ~ 53 months	Distance to nearest railway station, & neighbourhood population density & intersection density.	Pedometer Measured post-intervention (i.e., accumulated PA) Domain(s): Non-specific PA type(s): Steps	Participants living furthest from *a railway station* (quartiles 1) undertook fewer daily steps than those living closer (quartile 2, 3 & 4) in spring (differences = − 289,- 464, & -400, all p < .0001), summer (differences = 286, 498, 515, all *p* < .0001), autumn (differences = − 277, − 436, & -386, all p < .0001), & winter (differences = − 268, − 426, & -363, all p < .0001). *[* ***Amplification*** *]* Participants living in neighbourhoods with lowest *population density* (quartiles 1) undertook fewer daily steps than those in higher density neighbourhoods (quartiles 2, 3 & 4) in spring (differences = − 126, − 232, & -354, all *p* < .05), summer (differences = − 173, − 321 & -433 all *p* < .001), autumn (differences = − 154, − 272, & -378, all *p* < .01), & winter (differences = − 145, − 255 & -345, all *p* < .01). *[* ***Amplification*** *]* There were no significant differences in daily steps based on neighbourhood intersection density quartile in any season. ***[Invariant]***
**Jilcott et al. (2017)** **[** 51**]**	Lenoir County, North Carolina, USA (rural)	Patients with high blood pressure, ≥18 years Non-probability sample Analytical *n* = 249	One-arm pre-post quasi-experiment All participants received intervention	Type: Individual or group counselling (*Healthy Heart Lenoir*) Exercise sessions: No Counselling: Yes Awareness/education: Yes Group-based activities: Yes Length: 4 months (1 session/month)	1600 m network buffer around home. Walkability index (Walk Score®), density & distance to closest PA venues (parks, trails, & gyms) & food environment	Self-reported activity logs, & pedometer. Measured at baseline & 6-months Domain(s): Non-specific PA type(s): Total PA Steps	Increased distance (miles) to *private gyms* was associated with a larger increase in 6-month change in total PA (b = 31.7, *p* < .05) [***Suppression***] Increased density of *private gyms* was associated with a lower increase in 6-month change in daily steps (b = − 491.1, *p* < 05). [***Suppression*** **]**

*b* beta coefficient, *OR* odds ratio, *IRR* Incidence rate ratio

^a^Effect estimates presented were extracted from the original study and rounded

Several studies recruited participants from specific demographically-defined subpopulations such as African American, Hispanic or Latino [38, 44], low socioeconomic position [34, 47, 48], and males [40] or females only [38, 39, 44]. The target age of participants varied with some studies sampling older adults only [33, 37, 41, 47], and the remainder including samples representative of multiple age groups (e.g., young, middle-aged, and older adults). Three studies recruited some [32] or all [39, 51] participants from rural areas while all other studies recruited participants from urban areas. The analytical sample sizes ranged from 45 to 47,233 participants and all but five studies [40, 41, 45, 49, 50] applied non-probability sampling to recruit participants (e.g., convenience or volunteer sampling).

### Study designs

Thirteen studies reviewed included multi-arm experiments [32–44] and seven included single-arm experiments [45–51]. Among the multi-arm experiments, two [34, 40] included a three-arm intervention design while the remainder employed a two-arm intervention design. All but one multi-arm experiment [44] randomly assigned participants to treatment and comparisons arms. Eleven of the thirteen multi-arm studies estimated statistical interactions between BE variables and study arm (treatment vs. comparison) to determine whether the BE moderated intervention-facilitated PA. Despite undertaking multi-arm experiments, Hays et al. [35], Robertson et al. [43], and Zenk [44] combined data from intervention arms (e.g., full and partial intervention) to obtain pooled estimates of association between the BE variables and intervention-facilitated PA. Among the seven single-arm experiments, only one tested for an interaction between the BE and intervention [45]. All single-arm studies estimated associations between the BE and intervention-facilitated PA.

Half of all studies reviewed included interventions that lasted at least 12 months [32, 33, 35–37, 44, 45, 47, 49, 50]. Apart from five studies that measured PA post-intervention (i.e., adherence or average PA accumulated throughout the intervention period) [44, 46, 47, 49, 50], all other studies measured PA at multiple time points (e.g., baseline and follow-up) during their interventions.

### Physical activity intervention characteristics

All interventions aimed to increase some component (i.e., frequency, duration, or volume) of total or moderate-to-vigorous PA (MVPA) such as walking for leisure, transport, or errands (Table 2). Three studies delivered interventions at the population level [45, 49, 50] while the remainder of interventions were delivered at the individual level. For instance, Barnes et al. [45] evaluated the effectiveness of a mass media campaign and Hino et al. [49] and Hino et al. [50] evaluated the effectiveness of a city-wide pedometer-facilitated walking program on changes in PA among individuals. The provision of written or verbal information (including feedback from wearable activity trackers) intended to improve PA awareness or knowledge or that offered behavioural strategies was the most common intervention approach [32–35, 37–40, 43–45, 48–51] followed by group and one-to-one counselling (e.g., motivational interviewing and check-ins) [32, 34–40, 42–44, 48, 51]. Over half of studies reviewed included interventions with group-based activities [32, 33, 35, 37–39, 41, 44, 47, 51]. While not considered to be a group-based activity, the pedometer-facilitated intervention examined in two studies [49, 50] provided participants an opportunity to compare their progress relative to other participants. Notably, exercise sessions (e.g., walking groups) were offered to participants in less than half of studies reviewed [38, 39, 41, 43, 44]. Some studies used wearable activity monitors to measure PA outcomes however, participant self-tracking of PA via activity monitors (e.g., pedometers) was not a commonly used intervention strategy [43, 46, 49, 50].

### Measurement of physical activity

Self-reports were the most common method for measuring PA, with 14 studies including self-report measures alone or in combination with the use of accelerometers or pedometers (Table 2). Accelerometers captured PA in six studies [32–35, 38, 39], pedometers captured PA in four studies [43, 46, 50, 51] and heart rate monitors were used in one study [44]. Few studies included PA outcomes related to a specific purpose such as for transportation or errands [37, 45], or leisure or recreation [33, 37, 40, 42, 47] while most studies estimated overall accumulated PA (e.g., total MVPA, total steps, and PA-related energy expenditure). Six studies examined self-reported walking as an outcome [33, 36–38, 41, 45]. Two studies included PA outcomes associated with intervention adherence including completing prescribed walks [44] and attending exercise classes [47]. One study included a measure of perceived relative change in steps resulting from the intervention [49]. Moreover, one study captured neighbourhood-specific PA (inside and outside the neighbourhood) however, the authors combined these data to estimate total PA [51].

### Measurement of the built environment

GIS procedures with existing spatial databases were commonly used to estimate BE characteristics (Table 2). Two studies included BE data collected via street audits [38, 43]. Most studies defined neighbourhoods using network or Euclidean polygon buffers with radii ranging from 250 m to 1600 m of a participant’s home location (i.e., egocentric buffers). Most studies also developed walkability indices from GIS-derived BE variables [32, 33, 36, 37, 40–42, 44, 45] and or used Walk Score® [35, 39, 42, 46, 51]. Several studies estimated proximity variables including home-to-destinations distances (e.g., [34, 44, 47–51]), the absolute or relative density of BE attributes such as footpaths, intersections, residential populations, and green space [32, 35, 47, 48, 50, 51] and or the mix or availability, proportion, or count of recreational and utilitarian land uses and destinations (e.g., [33, 40, 44, 47, 51]).

### Methodological quality

The maximum methodological score available was 27. The methodological quality of included studies was considered moderate-to-high (total score: mean = 20.9; standard deviation = 3.0; minimum = 17; maximum = 27).

### Summary of findings

#### Invariant effects of the BE on intervention-facilitated physical activity

Eight studies provided evidence suggesting that neighbourhood walkability (i.e., Walk Score® or other index) likely had no impact on intervention-facilitated PA [32, 37, 40–42, 44–46] (Table 2). For most of these studies, there appeared to be strong evidence that the BE did not impact PA resulting from intervention participation, however, in three studies the presence of an invariant effect of the BE on intervention-facilitated was probable given the totality of the evidence provided. Carter et al. [32] found a statistically significant interaction between walkability and intervention arm, but found no statistically significant within-arm associations between walkability and steps among adults at-risk of diabetes receiving usual clinical care or a group-based education program. King et al. [37] found that compared with controls (i.e., health education arm), older adults exposed to a center and home-based physical activity intervention reported decreases in walking for errands if they resided in low walkable neighbourhoods only. However, a general reduction in walking for errands in both intervention and control arms was observed in high walkable neighbourhoods. Barnes et al. [45] found a non-significant statistical interaction between walkability and intervention arm among adults exposed to a state-wide mass media campaign. They also found a significant increase in the likelihood of achieving sufficient PA and a decrease in the likelihood of any transportation walking among residents of low walkable neighbourhoods, yet similar changes in PA were also observed among adults in high walkable neighbourhoods.

Seven studies also found other BE variables that had minimal impact on intervention-facilitated physical activity (Table 2). Four studies that included BE measures reflecting the availability of land uses and destinations in the neighbourhood (e.g., land use mix, access to parks and recreational facilities), found no associations with intervention-facilitated PA [33, 34, 47, 48]. Moreover, Robertson [43] found that BE factor scores including a pathway features other than safety factor, a pathways safety features factor, and a roads and bus stop factor were not associated with steps during a 3-month pedometer-facilitated walking intervention. In addition, changes in steps resulting from a city-wide pedometer intervention were not found to be associated with distance to the nearest railway stations [49] nor with intersection density [50] in Japanese adults.

#### Amplification effects of the BE on intervention-facilitated physical activity

Eight studies found BE variables that potentially amplified the effectiveness of their physical activity interventions (Table 2). Colom et al. [33] found positive associations between walkability, residential density, and intersection density and daily minutes of accelerometer-PA among obese adults exposed to a 12-month education based intervention. Lee et al. [38] found a positive association between the number of neighbourhood traffic control devices and levels of post-intervention walking, and between the number of neighbourhood crossing-aids and level post-intervention total PA in African American, Latino, and Hispanic women exposed to a 6-month PA intervention involving group-facilitated sessions and personalized plans. Combining the results from the full and partial intervention arms, Robertson [43] found several BE factor scores (i.e., dangerous and busy roads, commercial and residential use, traffic signals and pedestrian signage, and indoor fitness facilities and calming features) to be favorably associated with step counts following exposure to a pedometer-facilitated walking intervention. After exposure to a city-wide pedometer intervention, Hino et al. [49] found that neighbourhood bus stop density was positively associated with a self-reported increase in steps and Hino et al. [50] found proximity to the nearest railway station and higher population density to be positively associated with recorded steps. Recreational facilities were also found to amplify the effectiveness of PA interventions. For instance, combining the results from the full and partial intervention arms, Zenk [44] found positive associations between the presence of a public recreation center with treadmill or indoor track or indoor shopping mall (within 5-miles of home) with adherence to a 12-month walking program among middle-aged to older African-American women. Moreover, Garmendia [47] found that increases in area of well-kept community greenspace in the neighbourhood was associated with older adults attending at least 24 group exercise sessions over a 24 month period. Among inner city adults at risk of diabetes, Hays et al. [35] reported a positive association between greenery (Normalized Difference Vegetation Index) and change in accelerometer-measured MVPA during a 24-month intervention involving counselling and group-based education sessions.

#### Compensation effects of the BE

Findings from two studies suggested that intervention-facilitated physical activity may be more effective among those in less supportive BEs (Table 2). After finding a statistically significant “time-by-arm-by-walkability” interaction, Kerr et al. [36] also found a significant increase in daily walking minutes among men exposed to a 12-month personalized physical activity intervention residing in low walkable neighbourhoods. Lo et al. [39] found a non-significant interaction (Walk Score®-by-arm) however, they also found that MVPA increased among those who were exposed to the full intervention (multi-component) and decreased in those exposed to the partial intervention among middle-aged women who resided in neighbourhoods with a Walk Score® of zero.

#### Suppression effects of the BE

Four studies found evidence that the BE may suppress the effectiveness of physical activity interventions [36, 38, 43, 51] (Table 2). For instance, negative associations were found between the number of neighbourhood amenities (e.g., public garbage cans, benches, and drinking fountains) with post-intervention walking and total PA among African American, Latino, and Hispanic women exposed to a 6-month PA intervention [38]. Daily walking minutes were found to have decreased among men exposed to a 12-month personalized physical activity intervention if they resided in high walkable neighbourhoods [36]. Increased distance to private gyms was associated with increases in self-reported total PA, while the density of private gyms in the neighbourhood was negatively associated with pedometer-determined steps among adults exposed to a PA intervention involving individual and group counselling [51]. A more favorable greenspace and recreational facilities factor score was negatively associated with step count among adults exposed to a 3-month pedometer-facilitated intervention (full and partial arms combined) [43].

## Discussion

With the exception of one narrative review [22], reviews to date summarizing evidence on associations between the BE and PA have not focused on intervention-facilitated PA (e.g., [14–17]). Our systematic review summarized evidence from studies estimating the relationships between objectively-measured neighbourhood BE characteristics and intervention-facilitated PA in adults. Our findings advance prior knowledge by providing preliminary evidence demonstrating that the BE has the potential to influence intervention-facilitated PA, and therefore needs more consideration in the design and implementation of PA interventions. In some cases, the neighbourhood BE has the potential to amplify and even constrain the effectiveness of PA interventions that target adults.

Findings of studies included in our review were heterogeneous. Invariant effects of the BE on intervention-facilitated PA were more common than other effects. Approximately 75% of included studies (*n* = 15) found evidence of at least one BE variable as neither positively nor negatively influencing the effectiveness of the PA interventions. Invariant effects of the BE were found in relation to walkability, land use or destination mix or proximity, transit access or availability, and connectivity. Interventions that provide structured or facility-based PA opportunities such as walking groups (e.g., [39, 41]) or walking prescriptions [44] may be impacted to a lesser extent by the BE than interventions that provide little structure to PA routines. PA interventions that are less likely to be constrained by the local BE, such as facility-based exercise classes (e.g., [39, 41]), may have success even when the built environment is unsupportive of PA. However, it should be noted that our review found exceptions where neighbourhood walkability did appear to modify the effect of interventions involving facility and home-based exercise on PA (e.g.,[37]).

Approximately 40% of studies (*n* = 8) included in the review provided evidence that supported a potential amplification effect, whereby a supportive BE variable seemed to augment the effectiveness of a PA intervention. Amplification effects of the BE were found in relation to walkability, land use or destination mix or proximity, transit access or availability, connectivity, population or residential density, and traffic-related safety features. While this finding is positive in that some interventions may have an even larger impact on PA when implemented in supportive neighbourhood BEs, it also means that when interventions are universally implemented (e.g., mass media health promotion campaigns), widening health inequalities have the potential to emerge due to intervention effectiveness being conditional on other factors, such as the BE [52, 53]. These inequalities may be more pronounced if socially disadvantaged or vulnerable populations are also sorted into neighbourhoods with less PA supportive infrastructure [54, 55]. Two approaches to reducing these inequalities include modifying neighbourhoods with less supportive BEs to make them more supportive of PA (i.e., reducing the inequity) or designing PA interventions tailored to those residing in neighbourhoods with less supportive BEs.

The intervention’s compatibility with the BE context is important to consider during intervention development and implementation [22]. Approximately 10% (*n* = 2) of studies reviewed found reported findings that suggested the presence of a compensation effect whereby the PA intervention appeared to mostly benefit those residing in neighbourhoods with less supportive BEs. PA interventions targeting adults residing in less supportive BEs could be one strategy to increase PA among those who may not have access to local neighbourhood resources or opportunities that are supportive of accumulating sufficient levels of daily PA. An unexpected finding was that almost 20% of studies (*n* = 4) provided evidence suggesting that PA interventions implemented in the presence of a supportive BEs could have the unintended consequence of reducing PA (i.e., suppression). Suppression effects were found in relation to walkability, availability of destinations (greenspace and recreational facilities) and the availability of neighbourhood amenities. This negative effect potentially reflects a mismatch between intervention components and the context in which the intervention is implemented [22]. This negative effect could also reflect the presence of unmeasured neighbourhood factors that may act to inhibit intervention-facilitated PA (e.g., crime or incivilities). Speculatively, it could also reflect PA substitution [56, 57], whereby PA undertaken by a participant residing in a supportive BE (e.g., higher transportation walking in a high walkable neighbourhood) is replaced by the PA promoted by the intervention (e.g., facility-based exercise classes) potentially resulting in a reduction in overall PA. Neighbourhood BEs should be assessed for potential barriers to physical activity prior to the implementation of PA interventions, and the interventions adapted or modified to assist participants overcome these barriers where necessary. Such modifications may be as straightforward as providing participants with maps or information about local areas highlighting physical activity supportive infrastructure or walking routes [58].

Our review findings support previous evidence suggesting that the neighbourhood BE might be more supportive of walking than other physical activities [14–17]. Most PA interventions reviewed did not require participants to undertake structured exercise programs, thus participant decision-making regarding PA frequency, duration, type, and location during the intervention was primarily self-determined. Not surprisingly, much of the PA being encouraged during interventions were of moderate intensity, including walking. The extent to which transportation walking was specifically encouraged during interventions could not be ascertained from the intervention descriptions, despite most of the BE variables (e.g., walkability, mix and proximity to destinations) examined including those that are typically more supportive of transportation versus recreational walking [14–17, 57]. Walking is among the most commonly reported PAs undertaken by adults [59, 60] and the neighbourhood (e.g., streets and sidewalks, and public parks) is a popular location for walking [61–64]. While studies clearly defined the geographical areas used to estimate the BE variables (e.g., buffers, administrative units), PA outcomes lacked context specificity – i.e., no studies estimated the effects of the neighbourhood BE on intervention-facilitated neighbourhood-based PA. Nevertheless, many interventions appeared to encourage PAs that would likely occur outdoors and proximal to home and therefore have the potential to be impacted by the neighbourhood BE.

Despite most studies having moderate-to-high methodological quality, there remains major limitations of studies undertaken to date. The recruitment of participants from small geographical areas (e.g., clinics) might have resulted in less variability in the BE measures thus making small associations between BE and intervention-facilitated PA difficult to detect [65]. With the aim of being inclusive and comprehensive, we included randomized and non-randomized experiments in our review. Assuming adequate sample size, randomized assignment to treatment and comparison arms should theoretically result in balanced covariates, including for BE variables and unmeasured reasons for residential selection. However, in non-randomized experiments where participants self-select into a treatment or intervention arm, it is possible their reasons for treatment choice could align with the participants awareness of the potential built barriers and facilitators in the neighbourhood (e.g., residing in a walkable neighbourhood could be a motivator for choosing to participate in a PA intervention). As previously noted, in many studies there was a conceptual mismatch between the PA promoted by interventions and the types of BE variables measured. Moreover, it is difficult to attribute the influence of the neighbourhood BE on intervention-facilitated PA when it is unclear as to how much of this PA is being undertaken inside neighbourhood. To gain more rigorous evidence regarding the BEs role in influencing the effectiveness of PA interventions, we propose that where feasible, studies should include the following design elements:

- Sample from neighbourhood clusters or strata that vary in their BE supportiveness (to maximize BE variation) but that are similar or balanced in terms of their composition (e.g., SES, age distribution).
- Recruit intervention participants using probability sampling.
- Randomly assign participants to treatment and control arms, or where random assignment is not possible, collect data on participation reasons for treatment choice and neighbourhood selection.
- Include multiple data collection points to determine if the neighbourhood BE impacts the intervention effectiveness in the early, middle, and later stages of implementation (e.g., adoption, adherence, and post intervention) and to assess if sustained changes in PA in the maintenance period after the intervention has ended are conditional on the BE.
- Align PA measures with the target behaviour(s) of the intervention and where possible include measures of context-specific PA (e.g., GPS combined with accelerometers).
- Estimate interaction effects between BE variables and intervention-facilitated PA and perform BE/treatment arm stratified analysis to examine effect modification using groups with sample sizes that provide sufficient statistical power.
- Avoid combining the treatment and control groups to obtain pooled estimates of association between the BE and intervention-facilitated PA unless the interaction between treatment arm and BE is non-statistically significant and the control group is exposed to a subset of intervention components that were also received by the treatment group.
- Strengthened causal inferences by triangulating the quantitative evidence with qualitative evidence to gain insights as to how and why participants interacted with their BEs during the PA intervention (e.g., see [19, 20]).

We acknowledge several limitations of our review. Our review focussed only on the objectively-measured BE however, several studies have found associations between perceptions of the BE and intervention-facilitated PA [36, 46, 66, 67]. Perceptions of the BE are associated with PA [68, 69] even when controlling for the objective BE [70], yet self-reports and objective measures of conceptually similar BE characteristics are often discordant [70, 71]. Moreover, perceptions of the BE could be directly or indirectly influenced by the intervention (e.g., counselling to overcome perceived barriers) making it difficult to ascertain the extent to which the objectively-measured BE is impacting intervention-facilitated PA. Our categorization of associations between the BE and intervention-facilitated PA as invariant, amplification, compensation, and suppression while informative is somewhat rudimentary and it is possible that in some cases these mechanisms co-occur. For example, an invariant relationship may reflect some co-occurrence of amplification in a high supportive BE group and compensation in a low supportive BE group. The nature of these relationships may be easier to determine in randomized multi-arm experiments that include both the estimation of statistical interactions and effect modification compared with studies involving a single treatment group or intervention arm, with no control group. Although this is not always the case, especially when significant statistical interactions between the BE and PA do not result in statistically significant stratified group associations (e.g., [32]) or visa-versa [45]. Our broad categorization of BE and intervention-facilitated PA relationships is intended to prompt researchers and practitioners to consider how the BE might positively or negatively influence the effectiveness of their PA interventions. We reported on four intervention components that were offered to participants (exercise sessions, counselling, awareness/education, and group-based activities) as these were components frequently included in the interventions reviewed however, PA interventions tend to be more nuanced, and these groupings may not fully capture the intervention delivered to participants. Future studies should consider exploring the interactions between more detailed intervention components, such as Behaviour Change Techniques [72], and the BE. A realist review [73] may also offer a useful approach for obtaining comprehensive information about intervention design and implementation (e.g., consulting with intervention authors and reviewing intervention protocols and intervention materials) and their impact on PA within the context of the BE. Previous studies have identified intervention, sociodemographic, and cognitive characteristics associated with PA maintenance [74–76] yet congruent with our review findings, there appears to be a dearth of evidence regarding the BEs role in supporting PA after the conclusion of interventions (i.e., the maintenance phase). Thus, our review only focused on changes in PA that occurred during the intervention implementation. Our review excluded studies that involved modification to the built environment and thus our findings may not generalize to multi-level PA interventions that include components that both target individuals (e.g., programs) and modify the built infrastructure [18].

## Conclusions

It is important to consider PA intervention design and implementation from a socioecological perspective and to identify external factors, such as the neighbourhood BE that may interact with intervention effectiveness. Our review provides novel, but preliminary, evidence suggesting the effectiveness of interventions on PA could be conditional on the neighbourhood BE and that the in some cases supportive neighbourhood BE can increase and even decrease intervention effectiveness. The design and implementation of PA interventions, especially those that offer less structure or guidance or challenge participants to undertake PA in outdoor settings (e.g., walking), should take into consideration the level of supportiveness of the BE. More rigorous experimental studies, designed specifically to test the modifying effect of the neighbourhood BE on intervention-facilitated PA, are needed.

## Supplementary Information

**Additional file 1.**

## Data Availability

Materials from the study are available for further analysis. Application to access these materials can be made by contacting Dr. Gavin McCormack, Cumming School of Medicine, University of Calgary (gmccorma@ucalgary.ca). Applicants must be qualified researchers and are required to submit a brief proposal outlining their request and intended use of the material.

## Abbreviations

BE: Built environment
PA: Physical activity
MVPA: Moderate-to-vigorous physical activity
GIS: Geographical information systems
GPS: Global positioning systems
